# The 2023 fatal dengue outbreak in Bangladesh highlights a paradigm shift of geographical distribution of cases

**DOI:** 10.1017/S0950268824001791

**Published:** 2025-01-07

**Authors:** Mohammad Nayeem Hasan, Mahbubur Rahman, Meraj Uddin, Shah Ali Akbar Ashrafi, Kazi Mizanur Rahman, Kishor Kumar Paul, Mohammad Ferdous Rahman Sarker, Farhana Haque, Avinash Sharma, Danai Papakonstantinou, Priyamvada Paudyal, Md Asaduzzaman, Alimuddin Zumla, Najmul Haider

**Affiliations:** 1 Department of Statistics, Shahjalal University of Science and Technology, Sylhet, Bangladesh; 2 Institute of Epidemiology, Disease Control and Research (IEDCR), Ministry of Health and Family Welfare, Dhaka, Bangladesh; 3 Birmingham City University, Birmingham, UK; 4 Management Information System, Directorate General of Health Service (DGHS), Ministry of Health and Family Welfare, Dhaka, Bangladesh; 5 Faculty of Health Sciences and Medicine, Bond University, Robina, QLD, Australia; 6 The Kirby Institute, University of New South Wales, Sydney, New South Wales, Australia; 7 UK Public Health Rapid Support Team (UK PHRST), Department of Infectious Disease Epidemiology and Dynamics, London School of Hygiene and Tropical Medicine (LSHTM), London, UK; 8 BRIC-National Centre for Cell Science, Pune, India; 9 School of Medicine, Keele University, Keele, Staffordshire, UK; 10 Institute for Global Health and Wellbeing, School of Medicine, Keele University, Keele, Staffordshire, UK; 11 Department of Engineering, University of Staffordshire, Stoke-on-Trent, UK; 12 Division of Infection and Immunity, Centre for Clinical Microbiology, University College London and NIHR-BRC, University College London Hospitals, London, UK; 13 School of Life Sciences, Faculty of Natural Sciences, Keele University, Keele, Staffordshire, UK

**Keywords:** Bangladesh, dengue outbreak, geographical shift, meteorological factors

## Abstract

In 2023, Bangladesh experienced its largest and deadliest outbreak of the Dengue virus (DENV), reporting the highest-ever recorded annual cases and deaths. Historically, most of the cases were recorded in the capital city, Dhaka. We aimed to characterize the geographical transmission of DENV in Bangladesh. From 1 January–31 December 2023, we extracted and analyzed daily data on dengue cases and deaths from the Management Information System of the Ministry of Health and Family Welfare. We performed a generalized linear mixed model to identify the associations between division-wise daily dengue counts and various geographical and meteorological covariates. The number of dengue cases reported in 2023 was 1.3 times higher than the total number recorded in the past 23 years (321,179 vs. 244,246), with twice as many deaths than the total fatalities recorded over the past 23 years (1705 vs. 849). Of the 1,705 deaths in 2023, 67.4% (*n* = 1,015) died within one day after hospital admission. The divisions southern to Dhaka had a higher dengue incidence/1000 population (2.30 vs. 0.50, *p* <0.01) than the northern divisions. Festival-related travel along with meteorological factors and urbanization are likely to have contributed to the shift of dengue from Dhaka to different districts in Bangladesh.

## Introduction

The world is confronting its largest recorded dengue outbreak, with over 6.5 million cases reported in 2023 and more than 12 million cases in 2024 [1]. A substantial proportion of these cases are concentrated in South America, and South and Southeast Asia. Bangladesh is a densely populated nation in South Asia with a population exceeding 172 million [2], and has consistently experienced recurring outbreaks of dengue fever, particularly during the monsoon season [3]. This mosquito-borne disease, transmitted primarily by *Aedes* mosquitoes, has emerged as a critical public health concern, with significant surges in infections reported in 2019, 2021, 2022, and 2023 [3]. Despite initiatives aimed at controlling the spread of the disease, the country’s high population density and limited healthcare infrastructure have presented substantial challenges to effectively mitigating the outbreaks.

In 2023, Bangladesh witnessed its most extensive and deadliest dengue outbreak on record, marked by the highest annual tally of cases and fatalities due to Dengue virus (DENV) infection [4]. While dengue is endemic in Bangladesh, with cases reported annually since 2000 [3], the scale of the outbreak in 2023 is staggering and alarming. Recent years have seen a concerning uptick in dengue cases in Bangladesh, with over 82% of the total cases (*n* = 202,425) and 69% of deaths (*n* = 550) reported in the past five years (2018–2022) [2]. Historically, most of the dengue cases in Bangladesh have been reported in urban areas, with a particular concentration in the capital city of Dhaka, [5] except in some years (e.g. 2019) [6]. Sporadic dengue cases were documented in Dhaka in the 1960s, preceding the significant outbreak in 2000 in major cities, including Dhaka, Chattogram, and Khulna [5, 7]. Serological studies conducted across the country demonstrated substantial spatial heterogeneity in seropositivity with seroprevalence ranging from as high as 88% in urban Chattogram to as low as 3% in rural Maulvibazar in Sylhet division [8]. In the capital city Dhaka, the seropositivity of DENV ranged from 36 to 85% [8]. A recent study investigated the 2022–2023 dengue outbreak in Bangladesh, analyzing its characteristics, spatial distribution, and contributing factors. Dhaka and Chittagong emerged as major epicentres with higher caseloads and mortality [9].

*Aedes aegypti*, the primary vector of DENV is known for its preference for urban and suburban environments [10]. Several factors contribute to this affinity for urban areas including the presence of artificial containers, human habitation and blood hosts, microclimate in urban areas, and adaptability [10]. On the other hand, *Aedes albopictus*, the second important vector of DENV exhibits a broader habitat range including rural and urban areas [11]. Several factors are likely to contribute to the persistence of these outbreaks, including rapid urbanization, inadequate waste management, and climatic conditions such as heavy rainfall, flood, and high humidity, which create optimal breeding environments for mosquitoes [3, 7, 12, 13]. As Bangladesh has recently experienced a country-wide distribution of dengue cases, it is important to understand the factors that affect the geographical distribution of dengue cases in Bangladesh. In this study, we aim to characterize the geographical transmission of DENV infection and identify factors affecting the dispersion of dengue cases in Bangladesh.

## Methods

### Data source

We collected publicly available data on all dengue cases and death records from 1 January to 31 December 2023 from the daily press release of the Management Information System (MIS) of the Ministry of Health and Family Welfare, Bangladesh [14]. The MIS defined dengue cases based on clinical symptoms (including fever and rash) and laboratory tests for IgM to DENV and/or nonstructural 1 protein (NS-1) of DENV [15]. The MIS collected data from 77 hospitals based in Dhaka city (20 public and 57 private hospitals) and the district hospitals of 63 other districts of the country including the hospitalized patients in tertiary care medical college hospitals [3]. We further collected anonymized individual patient data including age, sex, village/ward level address, and hospital stays from the MIS for the same period. We collected 3-hourly meteorological data on temperature, relative humidity, and daily cumulative rainfall from ‘Bangladesh Meteorological Department (BMD)’ over the period 2000–2023 from the meteorological stations located in divisional headquarters including Agargaon, Dhaka (Lat 23.46, Lon 90.23), Chattogram (Lat 22.16, Lon 91.49), Rajshahi (Lat 24.22, Lon 88.42), Rangpur (Lat 25.44, Lon 89.14), Sylhet (Lat 24.54, Lon 91.53), Barisal (Lat 22.45, Lon 90.20), Khulna (Lat 22.47, Lon 89.32), and Mymensingh (Lat 24.43, Lon 90.26). We drew an imaginary east-west line in the middle of Dhaka city to compare the incidence and weather pattern of the southern (Chattogram, Khulna, and Barisal) and northern divisions (Rajshahi, Rangpur, Mymensingh, and Sylhet). As Dhaka division is located centrally in Bangladesh, it was excluded from the southern or northern part.

### Descriptive analysis

We plotted the age and gender-wise distribution of cases. We also summarized the hospital stays for the death cases. The full details on the hospital stays for the cases who survived were not available.

### Relative increase of dengue cases by division

We have estimated monthly relative changes in dengue cases in each division. The relative changes (an increase or decrease) of a division of dengue cases for a month were estimated with the formula as shown below

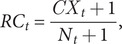

where *RC_t_* is the relative changes of dengue cases in *t* month, 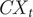 is the number of dengue cases reported in X city, and *N_t_* is the total number of cases in Bangladesh in *t* month. To avoid any complication of 0 cases in any city in any month we added 1 dengue case in both numerator and denominator.

### Incidence by district

We calculated the annual cumulative district-wise incidence of dengue cases by taking the cumulative annual number of dengue cases of each district divided by the population of the district shown as – (The total number of dengue cases in a district in 2023)/Total number of populations of that district) × 1000. We then generated a map for Bangladesh showing district-wise incidence of dengue cases in 2023. We compared the incidence by divisions (southern vs. northern).

### Statistical analysis

We compared the dengue cases and deaths for the year 2023 with the previous 23 years combined (2000–2022), created graphs, plots, and maps, and compared these data with meteorological parameters. We reshaped our dataset by incorporating division-wise outcome variables. We followed the list of districts for each division as shown in the daily dengue situation report shared by MIS [14]. We further collected division-wise population and geographical data from the Statistical Yearbook Bangladesh 2022 published by the Bangladesh Bureau of Statistics [16] including population size, the ratio of rural and urban population (which is a proxy variable for urbanization), and the distance of the district from the capital city, Dhaka. Additionally, we calculated population density by dividing the population size by the area of each district.

A generalized linear mixed model (GLMM) with a negative binomial distribution was used to model the outcome variable (division-wise daily dengue count), enhancing modelling flexibility through the inclusion of both fixed and random effects [17]. We introduced random effects into the GLMM to account for the longitudinal effects in the data [16]. The choice of negative binomial (NB) distribution allowed us to model response data appropriately with extra variations in the data (overdispersion) [18].

The components of the NB-GLMM are given below:

- Distribution: 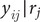 ~ Negative Binomial ( 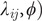 ,

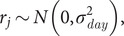

- Linear predictor: 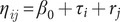

- Link function: 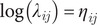 .

where 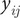 denotes the number of dengue cases in day *i* in division *j* (*i* = 1, 2, ⋯, 365; *j* = 1, 2, ⋯, 8), 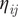 is the linear predictor, 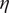 is the intercept, 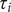 is the fixed effect due to day *i* for the *j*th covariate, and 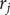 is the random effect due to division *j.*

The specific form of our model can be given by

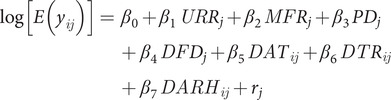

where 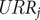 is the urban-rural ratio, 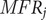 is the male-female ratio, 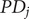 is the population density, and 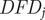 is the distance from Dhaka for the division *j*, 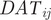 is the daily average temperature, 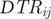 daily total rainfall, and 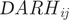 daily average relative humidity for day *i* and division *j.*

Parameter estimation in GLMMs is challenging due to the integration of random effects in the likelihood function [19]. The fixed effects (a measure of association), urban-rural ratio, male-female ratio, population density, distance from Dhaka (capital city), daily average temperature, daily total rainfall, and daily average relative humidity were used to estimate their impact on division-wise daily dengue counts (in number) and are expressed as incidence risk ratios (IRRs) with a 95% confidence interval (CI). Regarding the measures of variation (random effects), location with standard deviation (cluster), and intra-cluster correlation coefficient (ICC) were used. In addition, Akaike Information Criterion (AIC), Bayesian Information Criterion (BIC), Coefficient of Determination (R^2^), and Root-Mean-Square Error (RMSE) were used to report the variation of dengue cases at the division level and to test the goodness of fit of the model.

In our model, we used the daily division-wise dengue cases as the outcome variable which is a count variable, and the urban-rural ratio (as an urbanization proxy), male-female ratio, population density, distance from Dhaka, and several weather factors as the predictors. Variables with a *P*-value less than 0.05 in the final model were reported as statistically significant determinants of dengue cases [20].

## Results

### A record number of dengue cases and deaths in 2023

**Figure 1.**
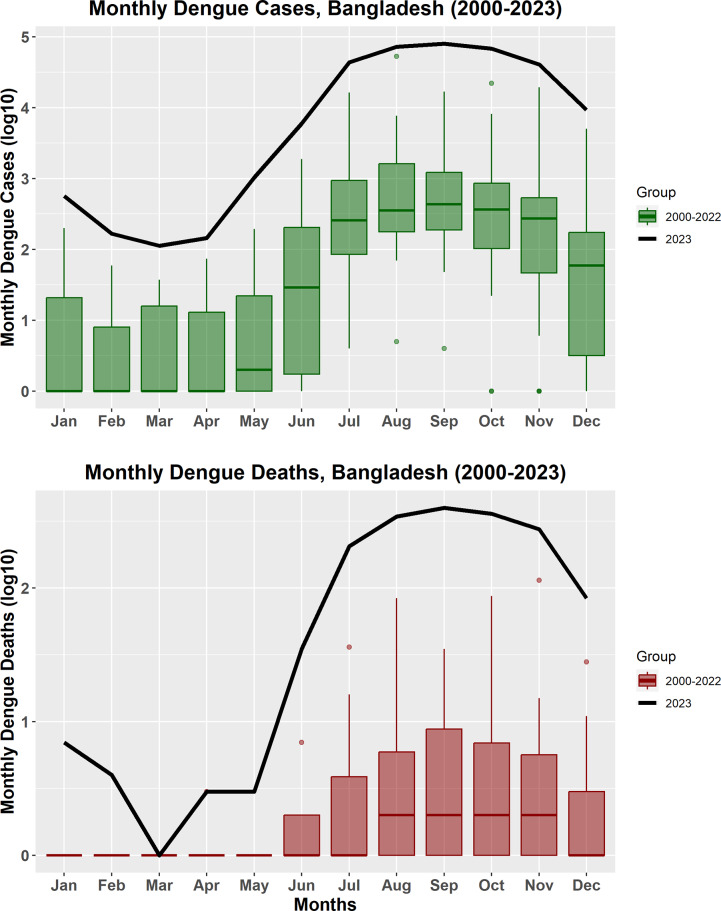
The total number of dengue cases and deaths reported in each month in 2023 compared to the total number of cases and deaths during the period from 2000 to 2022 in Bangladesh. Log 10 base is used to display the cases and deaths for the convenience of visualization and comparison.

During 2023 (1 January to 31 December), a total of 321,179 dengue cases were reported, resulting in 1,705 deaths and a Case-Fatality Ratio (CFR) of 0.53%. Between 2000 and 2022, Bangladesh reported a total of 244,246 dengue cases including 849 deaths, with a CFR of 0.35%. The number of cases reported in 2023 was 1.3 times higher than the total number of reported cases in the past 23 years (2000–2022): 321,179 vs. 244,246 and two times more deaths than the total number of fatalities recorded in the past 23 years: 1,705 vs. 849 (Figure 1). Among the individuals with dengue, 60% were male and 56% were below 30 years of age. A total of 110,008 cases were reported from the capital city of Dhaka including 980 deaths (CFR: 0.89%) while 211,171 cases were reported from outside Dhaka including 725 deaths (CFR of 0.34%). A higher proportion of cases were detected among young adults of <30 years (55 vs. 45%) but a greater proportion of deaths were detected among older adults of >30 years (68 vs. 32%) (Figure S1 in the Supplementary Material). Although males constituted a higher percentage of cases (60 vs. 40%) among total cases, females constituted a greater proportion of deaths (57 vs. 43%) among total deaths in 2023 (Figure S2 in the Supplementary Material).

**Figure 2.**
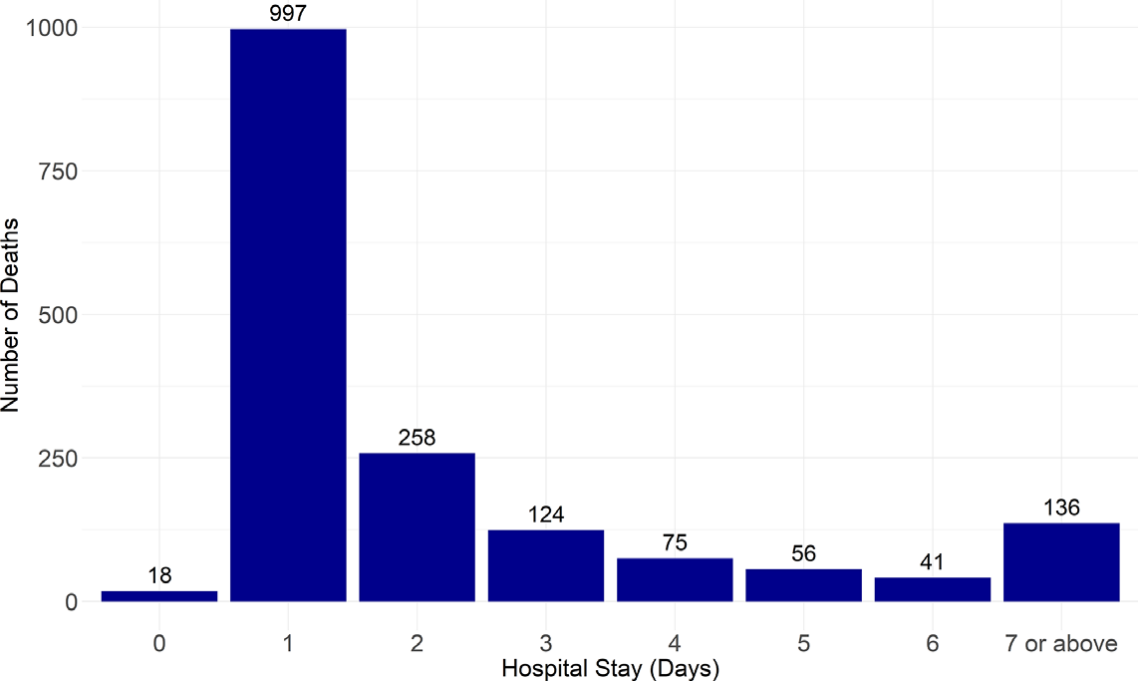
Number of days of hospital stays of 1,705 dengue cases in Bangladesh from 1 January to 31 December 2023. More than 67% (*n* = 1,015) of people died within one day of hospital admission.

Of the 1,705 people who died in 2023, 67.4% (*n* = 1,015) died within one day after hospital admission, with a mean hospital stay of 2.5 days (range: 0–61 days). The death toll increased to 74.6% (*n* = 1,273) in the first 2 days and reached to 81.9% (*n* = 1,397) in the first 3 days (Figure 2).

### Extended monsoon season in 2023

Bangladesh experienced a higher amount of rainfall in 2023 compared to the median annual rainfall for the period 2000 – 2022. The median rainfall for the period 2000 to 2022 was 1,843.1 (IQR: 257.10) mm whereas in 2023 total annual rainfall increased to 2,160.70 mm (Figure S3 in the Supplementary Material). In 2023, rainfall started earlier in the year with 75.8 mm of precipitation in March compared to a median of 29.5 mm amount of rainfall for the month for the period 2000 – 2022. There was a similar range of temperature between 2023 and the period 2000 – 2022 (mean of 28.25 °C [IQR:23.24-29.50 °C]) for the period 2000 – 2022 vs. mean of 27.06 °C [IQR: 25.15-29.93 °C] in 2023).

### Dengue cases and meteorological data in southern vs. northern divisions

The divisions southern to Dhaka had a higher dengue incidence compared to the northern divisions (2.30 vs. 0.50, *p* <0.01) per thousand population, whereas the central Dhaka division had an incidence of 2.90 per thousand population. In 2023, the southern divisions recorded slightly higher annual temperatures (27.46 vs. 26.54 °C) and also slightly higher relative humidity (80.79 vs. 79.08%) than the northern divisions (Table S4 in the Supplementary Material).

### Relative changes in dengue cases in each division

**Figure 3.**
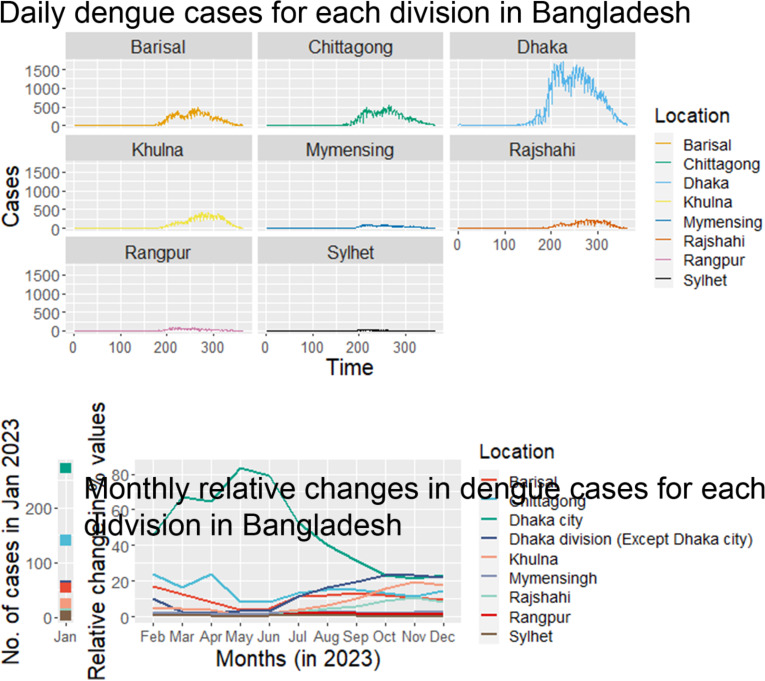
A (Top) Daily number of dengue cases in different divisions of Bangladesh (1 January–31 December 2023). B (Bottom). Monthly relative changes of dengue cases in each division in Bangladesh, 2023 from previous months. Although Dhaka city remains the centre of the outbreak, the percentage of cases has increased outside Dhaka city after July 2023.

Of the total 321,179 dengue cases in 2023, 211,171 (65%) were reported from outside Dhaka, whereas more than 57.5% (980 of 1,705) deaths were recorded in Dhaka. Dhaka city was the primary outbreak site in 2023 and contributed to more than 50% of the total cases up until July and then cases started to increase outside Dhaka, where Dhaka division (excluding Dhaka city) and Chattogram division have been among the prominent sites of the outbreak (Figure 3). The relative changes in dengue cases in different divisions became more evident after July when most divisions started to report an increased percentage of cases and Dhaka city started to report a lower percentage of cases (Figure 3). In November, the Dhaka division (except Dhaka city) reported almost 23% of dengue cases which was the highest number of dengue cases for any division in the country, the first record of surpassing the number of cases reported in Dhaka City by any division of the country (Figure 3). The Sylhet division contributed to less than 1% of cases throughout the year. The amount of annual total rainfall recorded in the northern divisions was 2,638.13 mm, as compared to 2,026.50 mm rainfall in the southern divisions (*p* < 0.01). The mean annual temperature recorded in the southern divisions was 26.60 °C, as compared to the 25.77 °C temperature of the northern divisions.

Increases in the numbers of dengue cases in both Dhaka city and outside were similar until mid-April. After that, dengue cases started to increase exponentially in the capital city Dhaka which continued up until the end of July 2023, and then the number of cases outside Dhaka surpassed the capital city. Notably, dengue-related deaths were initially higher outside Dhaka City until February, after which an escalation within Dhaka City commenced and persisted till the end of the year (Figure S5 in the Supplementary Material).

**Figure 4.**
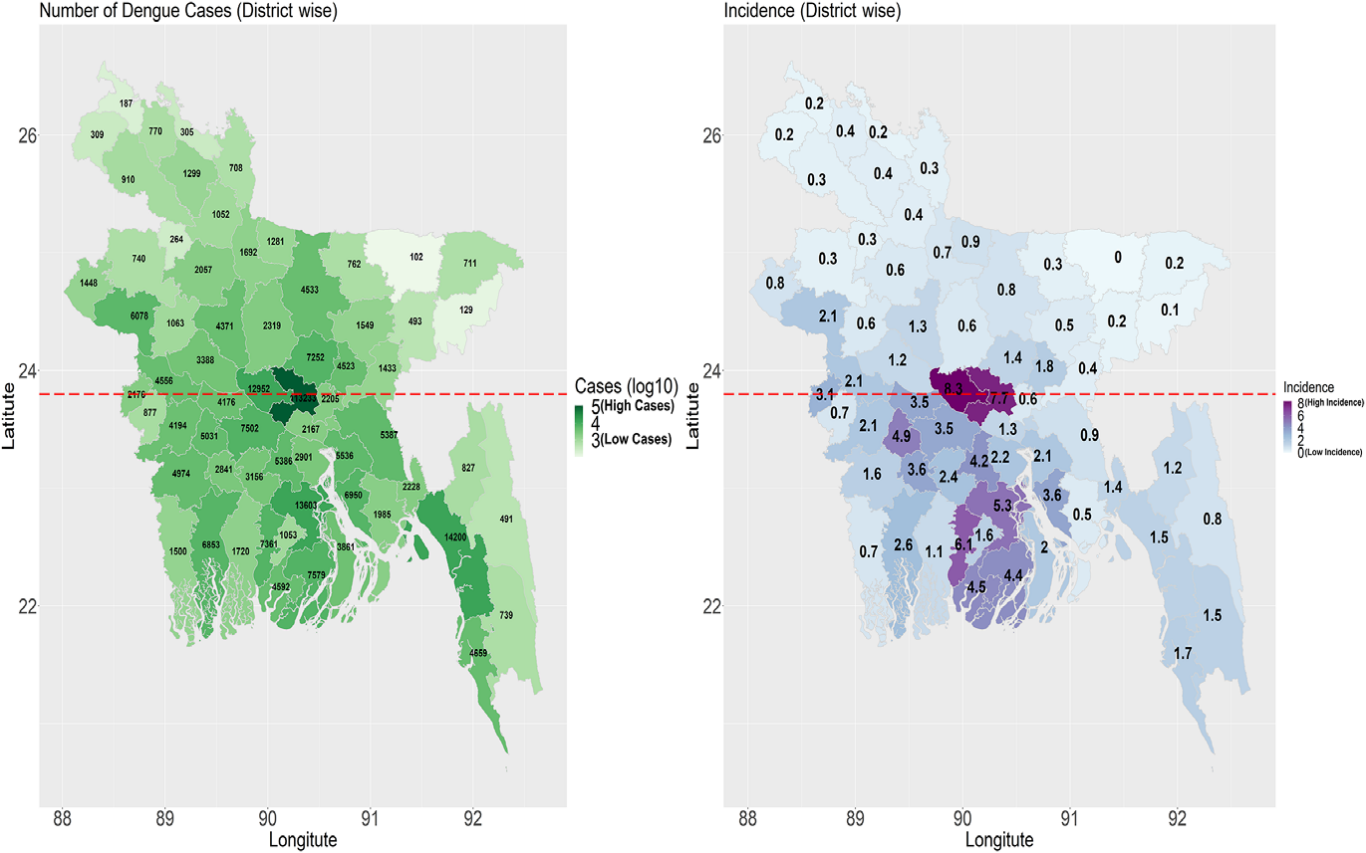
**A (Left).** Distribution of dengue cases in different districts of Bangladesh, 1 January 2023–2031 December 2023. **B (Right)** The incidence of dengue cases in each district in Bangladesh (1 January–31 December 2023). The horizontal line in the middle of the country divides the southern and northern divisions. The southern divisions (Khulna, Barisal, and Chattogram) have a higher mean incidence (2.30 vs. 0.50) and CFR (0.24 vs. 0.13) of dengue cases than the northern divisions. The southern division also had a higher annual mean temperature (27.46 vs. 26.54 °C) compared to the northern divisions in 2023.

District-wise, Dhaka district reported the highest number of dengue cases at 113,233, followed by Chattogram (14,200 cases), Barisal (13,603), Manikganj (12,952), and Patuakhali (7,579). On the contrary, the lowest dengue cases were recorded in Sunamganj (102), Maulvibazar (129), Panchagarh (187), Joypurhat (264), and Lalmonirhat (305). Dhaka district reported the highest death toll at 981, trailed by Barisal (167), Faridpur (138), Chattogram (106), and Khulna (41) districts (Figure 4).

### Correlation coefficient of dengue cases and deaths

For monthly dengue cases and deaths, positive correlations were observed between the population size of the district and the number of dengue cases (*r* = 0.44, *p* <0.001) and population size of the district and the number of dengue deaths (*r* = 0.43, *p* <0.001). A similar association is evident between population density and dengue cases (*r* = 0.47, *p* <0.001) and deaths (*r* = 0.43, *p* <0.001). A negative correlation was identified between the distance of each district from Dhaka city and the occurrence of Dengue cases (*r* = −0.32, *p* = 0.011) (Figure S6 in the Supplementary Material). We observed significant correlations between monthly dengue cases and various meteorological parameters in the divisions of Bangladesh, including average temperature (*r* = 0.13, *p* = 0.032), total monthly rainfall (*r* = 0.13, *p* = 0.025), and average humidity (*r* = 0.11, *p* = 0.052).

In the GLMM, a statistically significant positive association was identified between the dengue cases and daily average temperature (IRR: 1.13, 95% CI: 1.11–1.14), daily average relative humidity of the division (IRR: 1.09, 95% CI: 1.08–1.10), and urban and rural population ratio (IRR:1.04, 95% CI: 1.03–1.04). Daily total rainfall of the division (IRR: 0.99, 95% CI: 0.98–0.99), showed a significant negative association between dengue cases. Population density and distance from Dhaka also exhibited weak negative associations (Table 1).

**Table 1.** Factors associated with dengue cases in different divisions using a generalized linear mixed model between 1 January 2023 and 31 December 2023

Variables	Unadjusted IRR (95% CI)	*P*-value	Adjusted IRR (95% CI)	*P*-value
Urban–rural ratio	1.02 (1.02–1.03)	<0.001	1.04 (1.03–1.04)	<0.001
Male–female ratio	1.08 (0.97–1.21)	0.177	–	–
Population density	0.99 (0.98–0.99)	0.028	0.99 (0.99–1.00)	0.056
Distance from Dhaka (capital city)	0.99 (0.98–0.99)	<0.001	0.99 (0.98–0.99)	0.005
Daily average temperature	1.07 (1.06–1.08)	<0.001	1.13 (1.11–1.14)	<0.001
Daily total rainfall	1.01 (1.01–1.02)	<0.001	0.99 (0.98–0.99)	<0.001
Daily average relative humidity	1.07 (1.06–1.07)	<0.001	1.09 (1.08–1.10)	<0.001
**Groups Name**	**Variance**	**Standard Deviation**		
Location (Intercept)	0.002	0.050		
**Akaike information criterion (AIC)**	**Bayesian Information Criterion (BIC)**	**Root Mean Square Error (RMSE)**	**Coefficient of determination (*R***^**2**^**)**	**Intraclass correlation (ICC)**
23,720.90	23,774.80	181.80	0.435	0.002

IRR = Incidence Risk Ratio.

CI = Confidence Interval.

## Discussion

In 2023, the global death toll from DENV infection reached a historic high reporting of 7,000 annual deaths [20], with Bangladesh accounting for nearly one-fourth of these fatalities (*n* = 1,705). In addition to the high number of cases and deaths, the dengue outbreak in Bangladesh displayed several distinctive characteristics. There was a widespread distribution of cases across the country, extending beyond Dhaka. Notably, 67% of deaths occurred within the first 24 h of hospital admission. The CFR was exceptionally high in the capital city, Dhaka (0.89%) compared to the rest of the country (0.34%). Furthermore, the incidence of dengue cases was higher in the southern divisions compared to the northern divisions of Bangladesh. Dengue is a multifactorial disease. The reasons why Bangladesh observed such a large outbreak in 2023 needed detailed investigation.

A large majority of deaths (67%) occurred within the first day of hospital admission, suggesting severe disease and/or a considerable delay in seeking medical care. The precise cause of these deaths warrants thorough investigation. Below, we outline several possible explanations for the higher fatality rates observed on the first day of admission. Numerous patients likely arrived at the hospital with a delay. This may be related to the lack of awareness regarding dengue secondary infection and its complications. Hospitalized cases were likely to have composed of many secondary dengue infections. While primary dengue infection with one of the DENV serotypes tends to be mild and self-limiting, subsequent infection with another serotype may escalate to severe forms known as secondary dengue infection [21]. One key mechanism of secondary infection is antibody-dependent enhancement (ADE), where non-neutralizing antibodies increase disease severity [21]. Distinguishing between primary and subsequent dengue infection is often challenging, especially at the beginning of the illness when the symptomatology is similar. Thus, raising awareness regarding secondary dengue infection and promoting documentation or self-preservation of dengue test results in regions where health data are not recorded systematically is essential. Second, a significant portion of dengue patients (44%) [4] may have travelled to Dhaka from areas outside the capital city for treatment. These individuals either sought medical attention at a critical stage or were transferred after spending several days admitted to hospitals in districts or sub-districts, with those initial days not being counted as part of their final hospital admission. Many of these patients may have endangered their lives by undertaking long journeys to Dhaka without proper clinical management during the long journey. This may explain the higher number of deaths in the capital city. In Bangladesh, specialized medical care and management, including the facilities for Intensive Care Unit (ICU) beds, are mostly centralized in the country’s capital, Dhaka [4].

The sharp increase in dengue cases in 2023 is likely multifactorial including a higher temperature, humidity, urbanization, and population movement during the Eid festival. Dhaka is one of the most densely populated cities in the world with more than 22 million people living in approximately 300 square Kilometres, with a population density of 23,234 people/Km^2^ [22]. Many people travel to their rural homes during the two major religious festivals: Eid al-Fitr and Eid al-Adha. In 2023, Eid al-Adha was celebrated on 28 June. By this date, 76% of the 6,014 reported dengue cases had been recorded in the capital city, Dhaka [4]. In comparison, during the first six months of the year (January to June) from 2000 to 2022, an average of only 266 cases was reported nationwide [3]. The sharp rise in dengue cases in 2023 coincided with the Eid festival, which may have facilitated the spread of the virus as nearly 15 million people left Dhaka and surrounding cities (Gazipur and Narayanganj), to return to their rural homes [23]. This large population movement probably contributed to the spreading of the DENV throughout the county, as people infected with DENV can remain viraemic, and therefore, infectious for up to 12 days [24].

Another possible explanation for the increased dengue cases in 2023 may relate to species of *Aedes* mosquito species in different areas in the country. Although *A. aegypti*, the key vector of DENV transmission is a city-adapted mosquito, *A. albopictus*, is adapted more to rural settings. Earlier studies in Bangladesh reported the presence of *A. albopictus* in different parts of Bangladesh [8, 25]. In 2023, infected people travelling from Dhaka to rural areas may have spread the virus to the rural areas, where the *A. albopictus* mosquito maintained the local transmission [4]. Contrary to the popular notion of dengue being an urban disease, the significant number of cases observed in the rural areas of Bangladesh during the 2023 outbreak suggests that dengue might pose a substantial threat to rural communities in Bangladesh. The rural cycle of DENV transmission is usually led by *A. albopictus* which carries specific characteristics that make them a crucial vector for DENV. *A. albopictus* mosquito can bite non-human hosts, tend to bite outdoors, and breeds in tree holes and other natural settings, giving them better plasticity than *A. aegypti* [26].

Compared to the northern divisions, the southern divisions of Bangladesh had a higher incidence (2.30 vs. 0.50) and CFR (0.24 vs.0.13) of dengue cases in 2023. Although Bangladesh is a small country, there are some differences between the southern and northern parts of Bangladesh as districts in the southern parts observe higher rates of urbanization and population density. Also, the divisions in the south of Dhaka had 0.92 °C higher temperature (27.46 vs. 26.54 °C, *p* < 0.01) compared to the divisions in the north of Dhaka. Higher temperature has been associated with increased dengue cases because of its impact on the extrinsic incubation period of the virus and the increased biting rate of the mosquitoes [3, 27, 28]. However, it might be possible that a higher incidence of dengue cases in southern districts is an artefact of economic development in the regions which helped people visit healthcare facilities more frequently than their northern counterparts [29]. Our model also showed that the ratio of urban and rural population which we used as a proxy to indicate urbanization had an increased risk of having more dengue cases. We found a conflicting negative association between rainfall and dengue cases [28], which might be because of higher rainfall in the Sylhet division where the highest amount of precipitation is usually observed in Bangladesh. However, the relative humidity was positively associated with increased dengue cases in other countries including Thailand, the Philippines, and Sri Lanka [28].

In the past 23 years, Bangladesh recorded a CFR of 0.35% which is lower than the mean fatality rate in the South Asian region (1.9%) [30]. The CFR observed in Bangladesh in 2023 (0.53%) is 10 times higher than the World Health Organization’s (WHO) goal to limit the dengue-related CFR below 0.05%. [31]. In 2023, the CFR varied in different South Asian countries: 0.04% (20/51243) in Nepal, 0.09% (91/94198) in India, 0.06% in Sri Lanka, and 0.05% (1/1700) in Afghanistan (See the references in the appendix of Haider et al. [1]. The current dengue outbreak appears to continue into 2024, with Bangladesh reporting over 85,712 cases, 448 deaths, and a CFR of 0.52% as of November 23, 2024, [32]. In 2023, the CFR of DENV in Bangladesh may be elevated due to a particularly high fatality rate in the capital, Dhaka (0.91%). This increase may be linked to a higher incidence of secondary or tertiary infections, as the evidence suggests that over 80% of Dhaka’s population has previously been exposed to at least one DENV serotype [8]. Moderate to severe cases outside of Dhaka city have been referred and travelled to hospitals in Dhaka for better health care management.

To limit DENV infections in urban areas, particularly in Dhaka, it is crucial to regularly eliminate mosquito breeding sites and enhance surveillance for active cases [33]. Continuous monitoring of dengue cases will facilitate early detection and help to proactively identify hotspots. Public health authorities can then take swift action to control mosquito populations, isolate infected individuals, and launch public awareness campaigns on preventive measures [33, 34]. Early detection and prompt response are key to preventing the spread of dengue and mitigating its impact [33, 34]. Both construction management and residents should avoid storing water at construction sites or in homes during vacation periods. Additionally, it would be important to remain vigilant about early rainfall and rising temperatures, which can increase mosquito populations. Developing a municipal water system to reduce the need for water storage is essential for preventing Aedes mosquito proliferation [35]. Residents storing water for extended periods should take special precautions to avoid mosquito breeding [35].

Our study has several limitations. The data we presented in this study has been recorded through hospital-based passive surveillance in Bangladesh [14]. The surveillance covers a mere fraction (5%) of the country’s total healthcare facilities [4]. We did not have access to the circulating serotype data for the 2023 outbreak. However, several studies including WHO’s report on the Bangladesh dengue situation revealed that DENV-2 which reappeared in the country in 2023, became the predominant serotype (62%) along with DENV-3 (29%), and co-infection of DENV-2 and DENV-3 (10%) [36, 37]. Earlier, all four serotypes of the DENV have been recorded in Bangladesh at different times since 2000 [38, 39]. DENV-3 caused a larger outbreak in 2019 and remained a dominant serotype until 2022. DENV-4 reappeared in the year 2022 with co-circulation of DENV-1 and DENV-3 [38]. Thus, exposure to heterogeneous serotypes in 2023 likely to have increased the risk of severe dengue infection which has a much higher CFR than the primary infection [24]. While we observed significant differences in dengue incidence and CFR between the southern and northern divisions, potential biases linked to the passive surveillance method cannot be ruled out. While improbable, there’s a chance that district health officials in the southern division may have reported more diligently than those in the northern divisions, despite the reporting system being the same throughout the country.

## Conclusions

Bangladesh observed a large outbreak in 2023, with more than double the cumulative number of deaths than in the previous 23 years. Compared to the mean CFR of the past 23 years (0.35%), Bangladesh recorded a higher CFR of DENV in 2023 (0.53%). A large proportion (67%) of deaths was recorded within one day of hospitalization. A major geographic shift in dengue cases was observed, moving from the capital city, Dhaka, to the southern division in 2023. The transmission of dengue cases was likely to have been facilitated by urbanization, and higher temperatures, humidity, and lower rainfall in the southern districts. Improved estimation of mild or subclinical cases, their associated risk factors, and temporal trends are essential for implementing effective public health interventions. Contrary to the idea of dengue being an urban disease, our study shows that dengue poses a significant threat to rural communities in Bangladesh.

## Supporting information

Hasan et al. supplementary material Hasan et al. supplementary material

## Data Availability

The dengue case and mortality data presented in this study were obtained from publicly available press releases issued by the Ministry of Health and Family Welfare, Bangladesh (https://old.dghs.gov.bd/index.php/bd/home/5200-daily-dengue-status-report). Additionally, patient death records and hospitalization data were sourced from the Management Information System of the Ministry of Health and Family Welfare, Bangladesh. Meteorological data, including temperature, rainfall, and humidity, were acquired from the Bangladesh Meteorological Department through the Institute of Epidemiology, Disease Control and Research (IEDCR) as part of an ongoing collaboration. Bangladesh’s division-wise population and geographical data, such as population size, the rural-to-urban population ratio, and the distance of districts from the capital, Dhaka, were collected from the *Statistical Yearbook of Bangladesh 2022*, published by the Bangladesh Bureau of Statistics. The full dataset is available upon request from the corresponding author.

## Supplementary material

For supplementary material accompanying this paper visit http://doi.org/10.1017/S0950268824001791. click here to view supplementary material
