# Supplementary material for: The 2023 fatal dengue outbreak in Bangladesh highlights a paradigm shift of geographical distribution of cases
Source: Epidemiol Infect. 2025 Jan 7;153:e3. doi: 10.1017/S0950268824001791 (PMC11704938; doi:10.1017/S0950268824001791)

**Supplementary materials**

**S1. Fig: The age structure of dengue cases during 1 Jan-31 Dec 2023 in Bangladesh.** A higher proportion of cases were detected among young adults (<30 years) [55% vs. 45%] but a greater proportion of deaths were detected among older adults (>30 years) (68% vs. 32%).


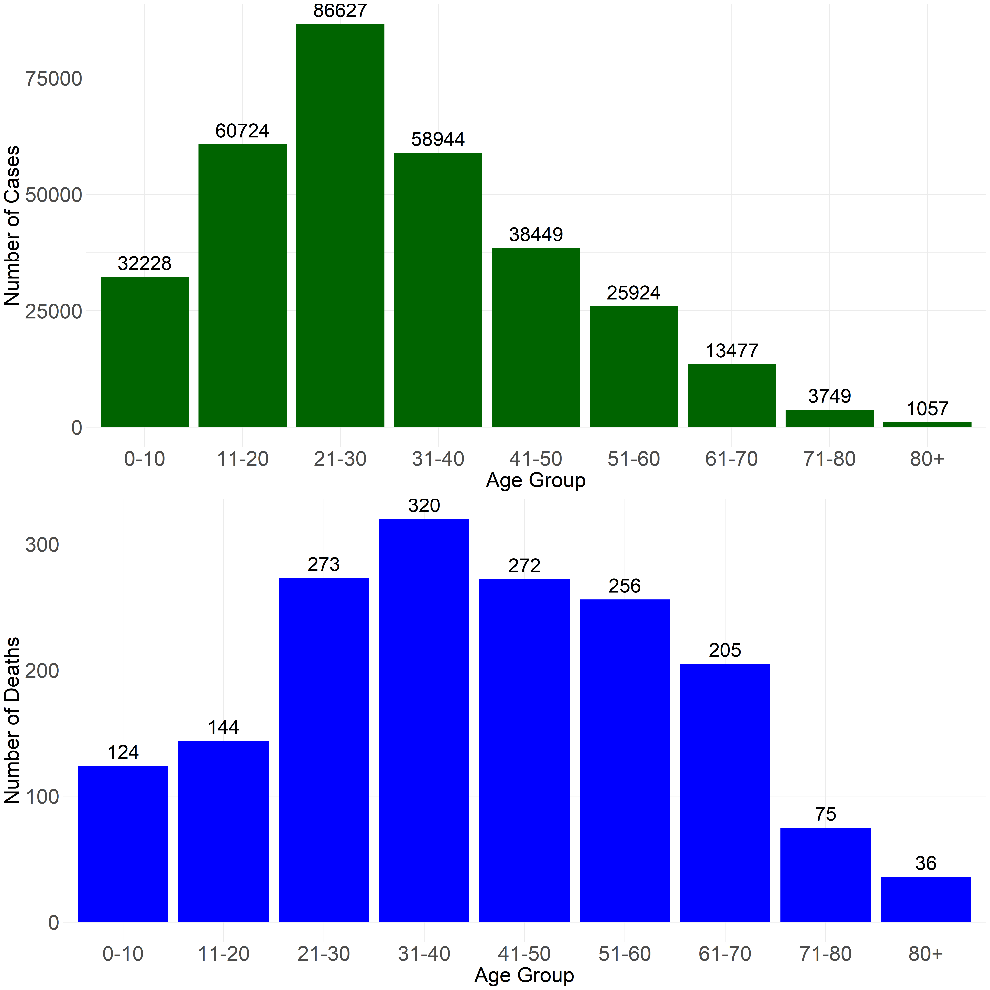


**S2. Fig. The comparison of the proportion of dengue cases and deaths in 2023 in Bangladesh by gender.** Although Males constitute a higher percentage of cases, females constitute a greater proportion of deaths.


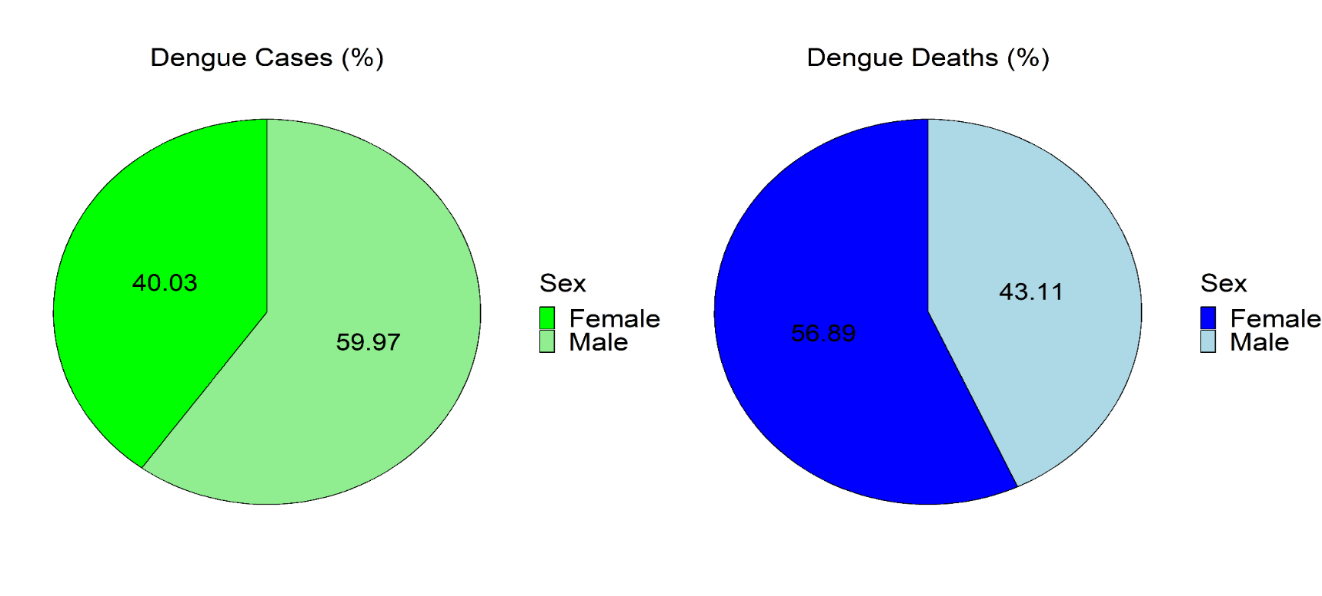


**S3. Fig.** The rainfall (mm) and temperatures (°C) recorded in a weather station in Agargaon, Dhaka, by Bangladesh Meteorological Department, Bangladesh for the period 2000-2022 vs. the rainfall in different months of 2023. Extended monsoon season was observed in 2023 in Bangladesh.


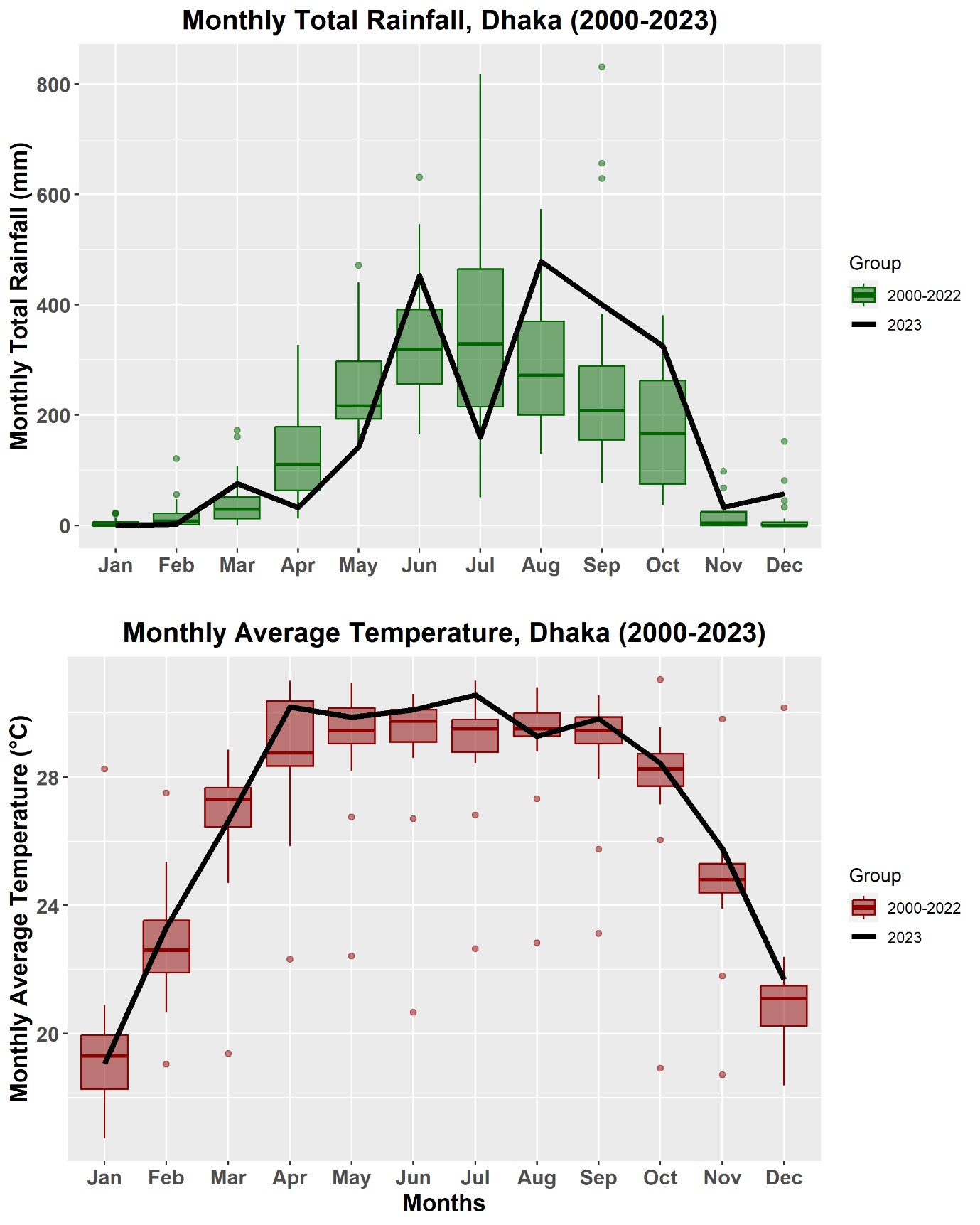


**S4. Table. The incidence and case-fatality ratio of dengue and annual temperature and rainfall in 2023 in the Southern and Northern divisions of Bangladesh**

| **Parameters** | **Southern divisions** | **Northern divisions** | **p-value** | **Dhaka division** |
| --- | --- | --- | --- | --- |
| Annual mean temperature (°C) in 2023 | 27.46 | 26.54 | <0.01 | 27.07 |
| Annual total rainfall (mm) in 2023 | 2026.5 | 2638.13 | 0.049 | 2160.7 |
| Annual mean relative humidity (%) in 2023 | 80.79 | 79.08 | <0.001 | 70.88 |
| Incidence of dengue (per 1000 population) in 2023 | 2.30 | 0.50 | <0.01 | 2.92 |
| Case-fatality ratio of dengue (%) in 2023 | 0.24 | 0.13 | 0.110 | 0.29 |

**S5. Fig.** The line graph of dengue virus infection in the capital city Dhaka and outside from 1 January to 31 December 2023. A large number of people from the capital city left Dhaka when Eid-Al-Adha was celebrated on the 28^th^ of June and subsequently, dengue cases started to increase outside Dhaka.


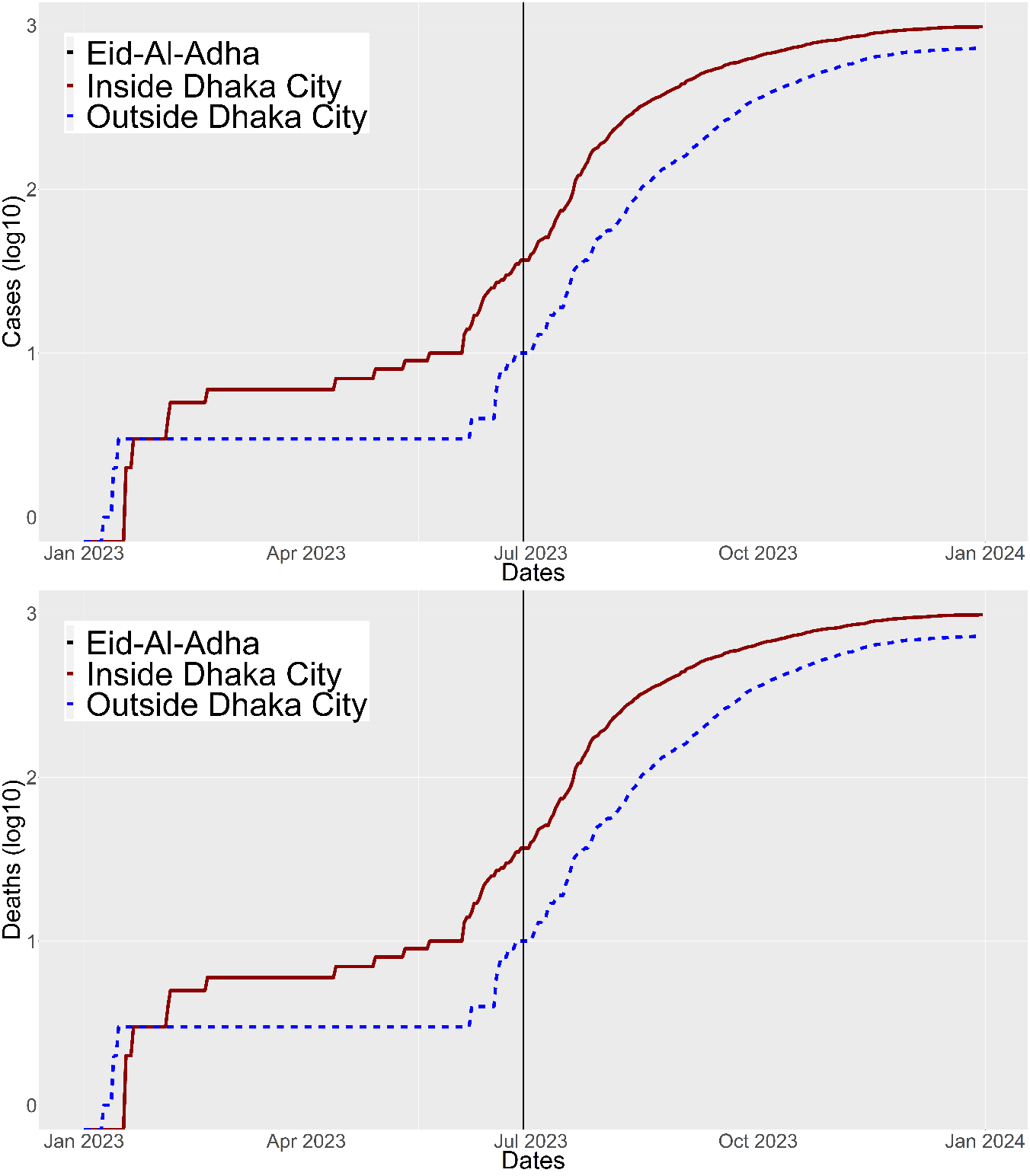


**S6. Fig. The correlation coefficient of dengue cases and deaths in different districts and their population size, population density, and distance from Dhaka city.** A positive correlation exists with the population density of the district and a negative correlation exists with the distance from the capital city Dhaka.


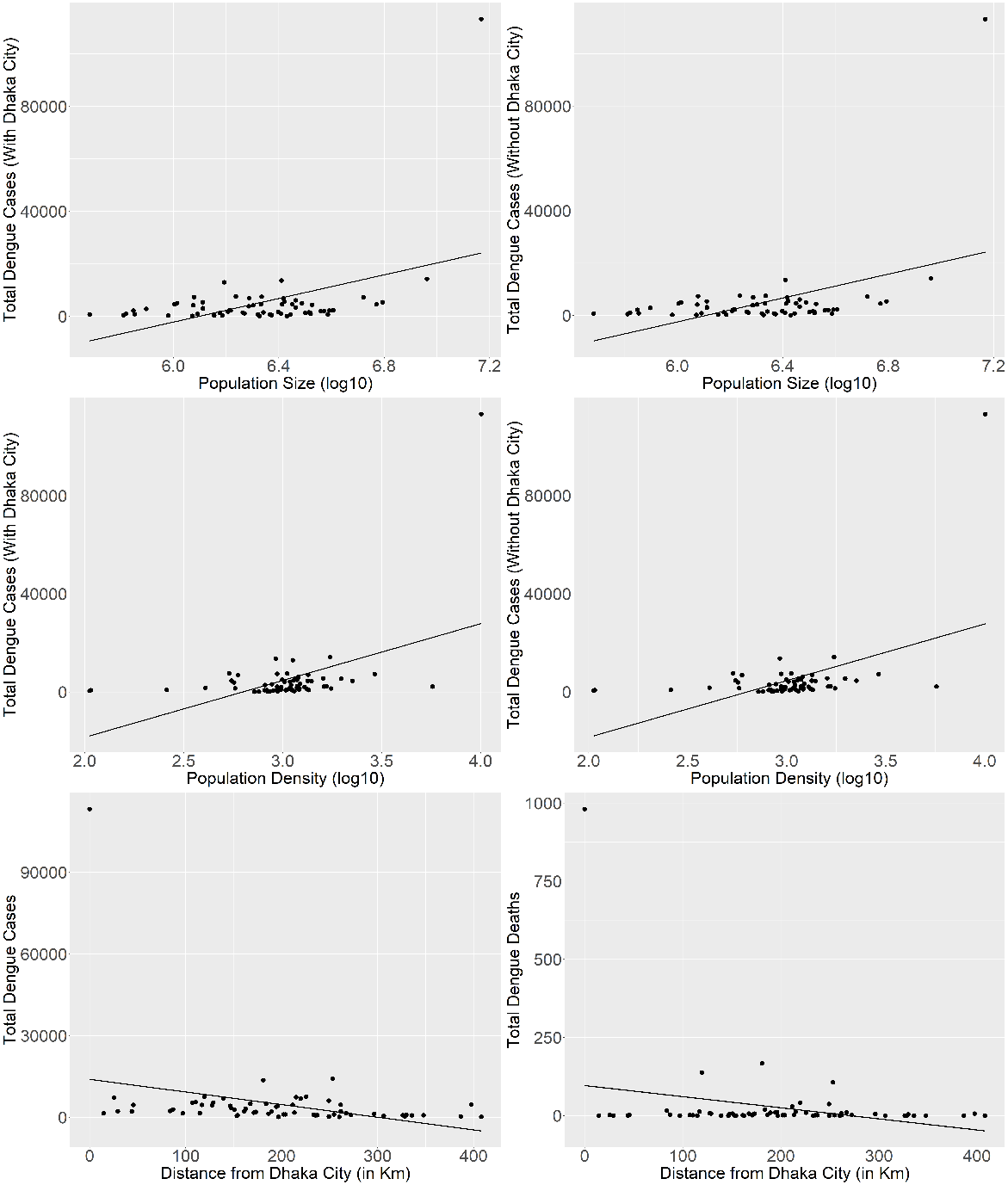

Supplement: Hasan et al. supplementary material [file S0950268824001791sup001.docx]
